# Seroprevalence and associated factors of Hepatitis B virus infection among pregnant women attending Antenatal care clinic in public hospitals in the Central Ethiopia region: A cross-sectional study

**DOI:** 10.1371/journal.pgph.0003921

**Published:** 2025-06-03

**Authors:** Yilma Markos Larebo, Abebe Alemu Anshebo, Sujit Kumar Behera, Natarajan Gopalan

**Affiliations:** 1 Department of Epidemiology and Public Health, School of Life Science, Central University of Tamil Nadu, Thiruvarur, India; 2 Department of Epidemiology, School of Public Health, Wachemo University, Hossana, Ethiopia; 3 Department of Midwifery, School of Nursing, Wachemo University, Hossana, Ethiopia; PLOS: Public Library of Science, UNITED STATES OF AMERICA

## Abstract

Hepatitis B virus infection poses a significant public health challenge among pregnant women in sub-Saharan Africa, including Ethiopia, where it is often underdiagnosed and underreported. This study aimed to determine the seroprevalence and associated factors of hepatitis B virus infection among pregnant women attending antenatal care in public hospitals in the Central Ethiopian region. A hospital-based cross-sectional study was conducted from October 1, 2023, to March 1, 2024, among 482 pregnant women selected using systematic random sampling. Blood samples were collected and tested for hepatitis B surface antigen, and data were gathered using a structured, interviewer-administered questionnaire. Data were entered into Epi Data version 3.1 and analyzed using SPSS version 26. Logistic regression analysis identified factors associated with hepatitis B infection, with significance at p < 0.05. The response rate was 98.8%. Most participants (66.2%) were aged 18–28 years, with a mean age of 28. The overall seroprevalence of hepatitis B virus infection was 12.8% (95% CI: 10.1, 16.2). A maternal educational level of diploma and above (AOR: 0.23; 95% CI: 0.09, 0.62) and no history of hospital admission (AOR: 0.23; 95% CI: 0.10, 0.53) were linked to a lower risk. In contrast, being unable to read and write (AOR: 2.67; 95% CI: 1.14, 6.26), having a medium (4–6) or large (≥7) family size (AOR: 2.34; 95% CI: 1.15, 4.78) and (AOR: 3.65; 95% CI: 1.33, 10.04), respectively, history of traditional delivery care (AOR: 2.46; 95% CI: 1.04, 5.84), and history of abortion (AOR: 2.90; 95% CI: 1.37, 6.11) were associated with higher risk. Hepatitis B virus infection remains highly prevalent among pregnant women in the study area. Strengthening family-centered healthcare, improving screening and vaccination coverage, and enhancing community-based awareness and prevention initiatives are essential to reduce the burden of infection.

## 1. Background

Hepatitis is a medical condition characterized by liver inflammation [1,2]. There are currently five hepatitis viruses worldwide, known as types A, B, C, D, and E [3]. They endanger the health of millions of people around the world [2,4]. According to the World Health Organization (WHO) reports, the estimated number of deaths from viral hepatitis increased from 1.1 million in 2019 to 1.3 million in 2022 globally, with 83% of deaths caused by hepatitis B and 17% caused by hepatitis C. Every day, 3,500 people die globally due to hepatitis B virus (HBV) and hepatitis C virus (HCV) infections [5]. HBV, a deoxyribonucleic acid (DNA) virus belonging to the hepadnavirus family, is the second leading cause of cancer-related deaths worldwide [6]. Approximately one out of fifteen people is affected by HBV [7].

Globally, an estimated 296 million individuals live with chronic HBV infection [8], with the African region accounting for a substantial proportion of this burden [9,10]. More than 75% of HBV infections occur in Asia, the Middle East, and Africa [11]. Chronic infection rates contribute to 26% of liver cancer cases and result in 19 deaths per 1,000 individuals [12]. Among pregnant women in this region, the prevalence rates of HBV infection vary. For instance, the prevalence is 7.5% in Sudan [13], 9.3% in Kenya [14], 3.2% in Eritrea [15], and 3.1% in Rwanda [16]. HBV infection remains prevalent in many low-income countries [17], with the mortality rate potentially reaching up to 25% [18].

In Ethiopia, there is a significant epidemic of viral hepatitis B, with a prevalence rate ranging from 8% to 12% [19,20]. The prevalence of hepatitis B surface antigen (HBsAg) is also high, ranging from 6% in adults to 12% in children, respectively [1]. Despite the considerable disease burden and potential for silent transmission, HBV is not widely recognized as a critical public health threat in many developing countries, including Ethiopia [21,22].

Mother-to-child transmission (MTCT) of the HBV during birth or early childhood infection within the first five years of life through contact with infected blood represents the primary mechanism of disease in areas with high endemicity [2,23]. In pregnant women, HBV can result in severe complications, including organ failure, postpartum hemorrhage, coagulation abnormalities, stillbirths, neonatal fatalities, as well as acute and chronic liver disease and hepatocellular carcinoma [24–26].

Several factors have been identified as potential contributors to the transmission of infections, including needle-stick injuries, circumcisions, tattoos, piercings, intravenous drug use, contact with sharp objects, transfusion of contaminated blood or blood products, unprotected sexual contact, and hemodialysis, which are considered forms of horizontal transmission [12,24]. Behavioural factors that increase the risk of transmission include alcohol consumption, frequent intercourse, and chewing Khat [12].

HBV is a small, tired DNA virus that reacts in hepatocytes, both acute and chronic liver disease [27]. Although no obvious treatment exists, effective antiviral agents such as tenofovir anticipate viral replication and reduce the risk of cirrhosis and hepatocellular carcinoma [28,29]. WHO recommends a comprehensive strategy for HBV control, including universal infant vaccination, timely birth doses, safe injection exercises, regular blood screening and diagnostics, and antiviral therapy [30]. These interventions have significantly reduced the transfer rate [31]. However, in low-resource settings, limited awareness, weak health infrastructure, and policy implementation intervals prevented the progress of the elimination of HBV [32,33].

Furthermore, limited studies have been conducted to investigate HBV infection and associated risks among pregnant women in this region. The primary objective of this study was to determine the seroprevalence and associated factors of hepatitis B virus infection among pregnant women attending antenatal care (ANC) clinics in public hospitals in the Central Ethiopian region. Finally, the result of this study will assist decision-makers in formulating strategies for pregnant women, infants, and healthcare professionals. It provides insights for global interventions, enhances the understanding of HBV, and supports screening, vaccination, and treatment efforts to reduce maternal complications and neonatal transmission, thereby contributing to international initiatives to eliminate HBV.

## 2. Methods and materials

### 2.1. Ethics statement

The Institutional Review Board (IRB) of Wachemo University (WCU) has diligently reviewed and approved the study, with Ethical Approval protocol Number: WCU-IRB 0022/2023, dated 06/03/2023. Before commencing the investigation, permission was obtained from the Central Ethiopia Regional Health Bureau, the Zonal Health Department, and the hospitals involved. Furthermore, the hospital managers in each selected study hospital acquired a formal permission letter. All the pregnant women had to provide written informed consent forms before participating in the interviews and blood sampling. To maintain confidentiality, all the collected information was kept anonymous.

### 2.2. Study area, design, and period

A hospital-based cross-sectional study was conducted among pregnant women in five selected public hospitals in the central Ethiopia region from October 1, 2023, to March 1, 2024. The regional government seat is at Hosanna, a town in the Hadiya zone, 232 kilometers from Addis Abeba, Ethiopia’s capital city. The region had an estimated total population of 6,430,235, with 3,186,824 (49.56%) men and 3,243,411 (50.44%) women. The total estimated number of women of childbearing age is 1,498,245 (23.3%), and pregnant women account for 222,486 (3.46%) of the population in the region [34]. This region comprises seven zones and three special districts, with 1,656 public and private health facilities (two comprehensive specialized hospitals, five general hospitals, 21 primary hospitals, 228 health centers, 1,067 health posts, and 333 private clinics). The five selected hospitals offer antenatal care services to more than 39,730 women yearly, including 14,700 women in the past three months. All five facilities are well-equipped, have high patient volumes, and have regional representation. They also play a role as referral centers for maternal health services and offer comprehensive antenatal care services, such as hepatitis B virus screening (testing for hepatitis B surface antigen (HBsAg) to detect active HBV infection, with positive cases receiving counselling, referral, and management to prevent MTCT) and delivery services, including caesarean delivery [35].

### 2.3. Sample size determination

Using the single population proportion formula [36], the sample size was determined by considering the following assumptions: P = 11.6% [37] (where P represents the prevalence of HBV infection among the mothers in the Tigray Region), Z1-α/2 = 1.96 (where Z1-α/2 represents the critical value at a 95% confidence level), d = 3% (where d represents the margin of error), and a 10% non-response rate, $n\;=\;\frac{{\left(\frac{Z\alpha }{2}\right)}^{2}p\;(1-p)}{d^{2}}$ (where n = the desired sample size) n = (1.96)^2^ 0.116(1- 0.116)/ (0.03)^2^ and finally, by adding a 10% non-response rate during the study, n = 438 + 10% of 438, which was 44. Thus, the total sample size was 482 pregnant women, who were included after adjusting the non-response rate.

### 2.4. Population

All pregnant women visiting ANC clinics at public hospitals in the central Ethiopian region were considered the source population. In contrast, the study population was randomly selected from all pregnant women visiting ANC clinics at public hospitals in the central Ethiopian region.

### 2.5. Sampling procedure

**Fig 1:**
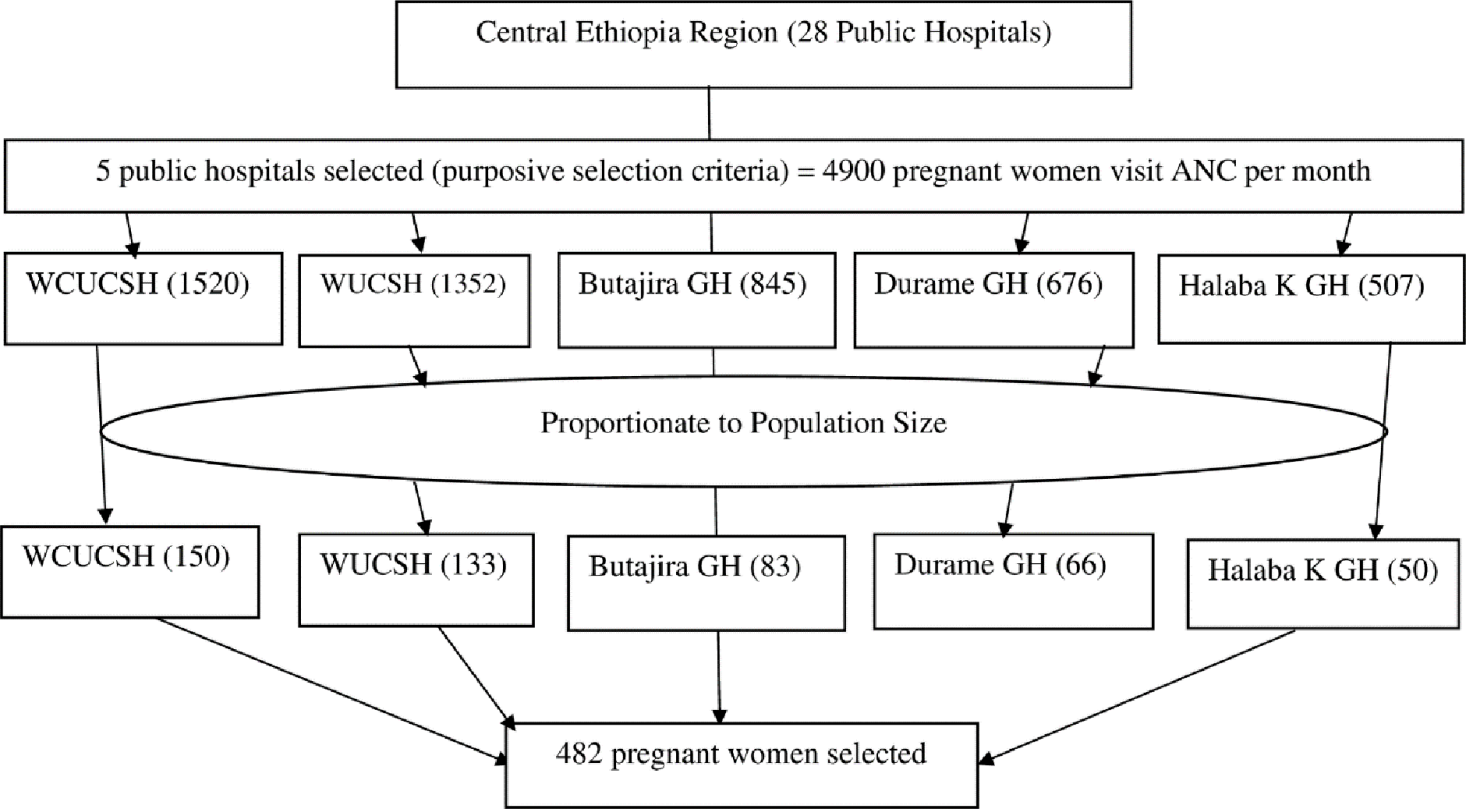
Schematic presentation of sampling procedure on seroprevalence of hepatitis B virus infection among pregnant women attending antenatal care clinics in public hospitals in Central Ethiopia region, 2023 (n = 482).

The study was conducted in five purposively selected public hospitals in central Ethiopia: two teaching hospitals, Wachemo University Nigist Eleni Mohammed Memorial Comprehensive Specialized Hospital (WCUCSH) and Worabe Comprehensive Specialized Hospital (WUCSH), as well as three general hospitals, Butajira Hospital, Durame Hospital, and Halaba Kulito Hospital. ANC registration data indicated that approximately 14,700 pregnant women visited these facilities over the past three months, resulting in an average of 4,900 monthly visits across the five hospitals. The sample size was proportionally allocated based on the ANC caseloads. The pregnant women in the hospital’s ANC clinics were chosen randomly as the index pregnant women for interview questions. Finally, the 482 pregnant women were chosen using a systematic random sampling technique with a skip interval of every 10^th^ (sampling interval k = N/n = 4900/482 ≈ 10). Data collection continued until the target 482 sample size was attained (Fig 1).

### 2.6. Inclusion and exclusion criteria

All pregnant women aged 18–49 who had lived in the study area for at least six months, visited the ANC clinic,and had confirmed pregnancies through clinical history,physical examination, or obstetric ultrasound scans were eligible for inclusion. Women who were severely ill and unable to communicate, as well as those who were diagnosed with human immunodeficiency virus (HIV) infection and started antiretroviral therapy (ART), who declined to provide informed consent, and who had resided in the area for less than six months, were excluded from the study.

### 2.7. Variables of study

#### 2.7.1. Dependent variable.

The serostatus hepatitis B virus infection (1: Yes, 0: No).

#### 2.7.2. Independent variables.

The predictor variables were as follows: Basic sociodemographic characteristics such as age in years, residence, marital status, educational status, occupation status, family size, average monthly income, and sources of information about HBV infection. Past medical history and behavioural risk factors assessed in this study included history of hospital admission, history of surgery, tonsillectomy, phlebotomy, history of blood donation, tattooing, body piercing, genital discharge, history of intravenous drug use, injectable medications, dental procedures, multiple sexual partners, unsafe sex, history of contact with hepatitis patients, history of mortality due to hepatitis in the family, previous history of emigration, history of sexually transmitted illness (STI), provision of traditional delivery care, presence of opportunistic infection, alcohol drinking, Khat chewing, and smoking cigarettes. Obstetric and clinical-related characteristics of the study participants, like previous history of ANC visits, history of previous delivery, place of the previous delivery, previous history of abortion, female genital mutilation (FGM) history, gestational age, parity, gravidity, HBV transmitted sexually, HBV transmitted through contact with blood and body fluid, HBV transmitted from mother to child, and other routes of transmission.

### 2.8. Operational definitions

A multiple sexual partner is a person who was engaged in sexual activity with two or more partners in 12 months [9].

Hospitalization history refers to the subject’s previous hospital admission history for any disease [38].

History of blood transfusion is related to the patient’s history of blood transfusion for any problem [38].

Phlebotomy is drawing blood from a vein using a needle for diagnostic testing, medical treatment, or research purposes [39].

Tonsillectomy is the surgical removal of the tonsils, commonly performed to treat recurrent throat infections [40,41].

Gestational age (GA) is the fetus’s age starting from fertilization [42].

Parity is the number of live-born children a woman has delivered[42].

(i) Primipara: those who gave birth only once
(ii) Multipara: those who gave birth above one time
(iii) Grand-multi: those who gave birth more than five times.

Family size is the number of household members, including the study participants with a small-sized family (1–3 members), a medium-sized family (4–6 members), and a large-sized family (≥ seven members) [43].

### 2.9. Data collection and quality assurance procedures

Before data collection, five Bachelor of Science (BSc) midwives were recruited as data collectors, and two BSc nurses were assigned as supervisors. Additionally, five BSc laboratory technologists working in the selected public hospitals were involved in the study. All personnel received two days of training on study procedures, collecting samples, testing HBV infection and transmission facts, participant counselling, and safety concerns/confidentiality. The training was conducted at Wachemo University, the center for All.

The data was collected using a structured interviewer-administered questionnaire. The questionnaire included questions from various literature sources [37,41,44–46]. Prior permission was obtained from the original owner of the questionnaire. The questionnaire was initially prepared in English and then translated into the common language, Amharic, to check the consistency of the items. It was then translated back to English to verify the accuracy of the Amharic translation. To maintain uniformity, the survey was pretested on a sample of 5% (n = 24) of individuals outside the study area through private interviews.

Additionally, two trained supervisors conducted rigorous daily reviews of the collected data to ensure data quality. They focus on assessing the data collection instrument for its simplicity, integrity, face validity, internal consistency, and completeness. Particular emphasis was also placed on the instrument’s clarity and ease of use and the application of standardized community rating scales.

### 2.10. Laboratory tests

During the ANC visits, three milliliters of venous blood were collected from selected pregnant women after proper patient preparation. Each participant was seated comfortably, and venipuncture was performed at the median cubital vein of the antecubital fossa using standard sterile techniques. To maintain infection prevention and control (IPC), data collectors practiced hand hygiene, used new sterile gloves, and employed single-use needles and vacutainer tubes for each participant. Blood was drawn into sterile vacutainer tubes containing anticoagulant (heparin), appropriate for plasma-based HBsAg screening.

Blood plasma was separated for hospital-based studies using an electric benchtop centrifuge. In the hospital setting, complete plasma separation using the benchtop centrifuge typically requires 10–15 minutes under standard conditions [39,47]. The collected blood plasma samples were screened for HBV (HBsAg) infections using the Guangzhou Wondfo Biotech assay test cassette, following the manufacturer’s guidelines. This one-step whole-blood plasma test cassette is a rapid immunochromatographic assay (ICA) produced in China and designed to detect HBV through HBsAg in human blood plasma qualitatively. The result is available in 15 minutes. It can be stored at temperatures of 4ºC to 30ºC. It is efficient to test 5 items at a time; it is simple to use, and no equipment is required to process the specimen and read the result. It is also visual, rapid, sensitive, and accurate. It has a sensitivity of 96.2% and a specificity of 99.3%, comparable to the results obtained with commercial test kits. The blood samples were added to the cassette according to the manufacturer’s instructions [48]. Finally, the results were interpreted based on seropositive (HBsAg+) presence, indicated by two distinct red bands in the test and control regions.

Conversely, seronegative (HBsAg-) is indicated by a single red band solely in the control region, and no apparent red or pink band is present in the test region. HBsAg is invalid if the control band fails to appear, which means an improper testing procedure or deterioration of reagents; therefore, the test should be repeated [9]. Finally, pregnant women who tested HBV (HBsAg+) were referred to internal medicine linkage programs for further investigation, prevention, treatment, and care services.

### 2.11. Data processing and analysis

Data were entered and validated EpiData version 3.1 (EpiData Association, Odense, Denmark) [49]. The dataset was reviewed for completeness, consistency, and accuracy before entry. Any inconsistencies or missing responses were cross-checked against the original questionnaires and corrected accordingly. Double data entry and verification procedures were employed to ensure data quality, and no missing values were observed in the final dataset. The cleaned and coded data were then exported to the Statistical Package for Social Sciences (SPSS) version 26.0 (IBM Corp., Armonk, NY, USA) for analysis [50].

Descriptive statistics summarized the study variables: frequencies, percentages, means, and standard deviations. Findings were presented using tables, graphs, and charts, as appropriate. Bivariable logistic regression analysis was first conducted to identify variables associated with HBV infection. Variables with a p-value less than 0.25 were considered candidates for the multivariable analysis.

Multivariable logistic regression analysis was conducted using a backward stepwise method to identify independent predictors of HBV infection. Adjusted odds ratios (AORs) with 95% confidence intervals (CIs) were reported, and statistical significance was set at a p-value < 0.05. The Hosmer and Lemeshow test evaluated the model’s fit, and the variance inflation factor determined whether there was multicollinearity among the independently associated variables.

### 2.12. Inclusivity in global research

Additional information regarding the ethical, cultural, and scientific considerations specific to inclusivity in global research is included in the Supporting Information (S2 Checklist).

## 3. Results

### 3.1. Sociodemographic characteristics of the study participants

Six of the 482 study participants eligible for inclusion were excluded due to a known history of HBV infection and having received HBV infection treatment. These excluded data sets were not considered for analysis, resulting in a response rate of 98.8%. Of the 476 study participants, 346 (72.7%) resided in urban areas; the majority (n = 315; 66.2%) were 18–28 years old. The mean age of the pregnant women was 28 ± 4.88 years, and most participants, 436 (91.6%), were married, and 213 (44.7%) had completed primary and secondary education. A total of 204 (42.9%) participants were unemployed, and more than half (246; 51.7%) of the participants came from small-sized families (1–3 members). Moreover, most 365 (76.7%) participants had access to information about HBV infection. More than half (259; 54.4%) of the study participants reported a low household income of ≤ 2000 ETB (≤ 34.74 USD) (Table 1).

**Table 1:** Sociodemographic characteristics of study participants.

		Total(n = 476)
Variables	Categories	n (%)
Age	18-28	315(66.2)
	29–39	150(31.5)
	≥40	11(2.3)
Residence	Urban	346(72.7)
	Rural	130(27.3)
Marital status	Single	14(2.9)
	Married	436(91.6)
	Ever married	26(5.5)
Educational level	Unable to read and write	62(13)
	Primary and secondary education	213(44.7)
	Certificate	84(17.6)
	Diploma and above	117(24.6)
Occupation	Employed	151(31.7)
	Self-employed	121(25.4)
	Unemployed	204(42.9)
Family size	Small family (1–3)	246(51.7)
	Medium family (4–6)	176(37)
	Large family (≥ 7)	54(11.3)
Information about HBV infection	Yes	365(76.7)
	No	111(23.3)
Average monthly income	≤2000ETB (≤34.74USD)	259(54.4)
	2001-4000(34.75-69.74USD)	38(8)
	>4001ETB (>69.75 USD)	179(37.6)

Where: HBV: Hepatitis B virus; ETB: Ethiopian Birr; USD: United States Dollars.

### 3.2. Epidemiological characteristics of study participants

Of the 476 study participants, 287 (60.3%) had a previous history of hospital admission, 60 (12.6%) study participants had a history of surgery, 74 (15.5%) had a tonsillectomy, 78 (16.4%) had a phlebotomy, and 39 (8.2%) had a blood donation. A total of 38 (8%) study participants had a history of tattooing, 174 (36.6%) had body piercings, 66 (13.9%) had genital discharge, 194 (40.8%) had intravenous drug use, and 49 (10.3%) had dental procedures. Approximately 46 (9.7%) of the study participants had a history of multiple sexual partners. Sixty-seven (14.1%) had unsafe sex, 25 (5.3%) had a family history of hepatitis, 84 (17.6%) had sexually transmitted infections, 105 (22.1%) had a previous history of receiving traditional delivery care, and 36 (7.6%) had the presence of opportunistic infections. Among the total study participants, 19 (4%) had a history of alcohol consumption, 68 (14.3%) had a history of Khat chewing, and 20 (4.2%) had a history of smoking cigarettes (Table 2).

**Table 2:** Past medical history and behavioural risk-related characteristics of study participants.

		Total(n = 476)
Variables	Categories	n (%)
History hospital admission	Yes	287(60.3)
	No	189(39.7)
History of surgery	Yes	60(12.6)
	No	416(87.4)
Tonsillectomy	Yes	74(15.5)
	No	402(84.5)
Phlebotomy	Yes	78(16.4)
	No	398(83.6)
History of blood donation	Yes	39(8.2)
	No	437(91.8)
Type of donation	Voluntary	39(100)
	Replacement	0(0)
Frequency of donation	First time	29(74.4)
	Repeated	10(25.6)
Tattooing	Yes	38(8)
	No	438(92)
Body piercing	Yes	174(36.6)
	No	302(63.4)
Genital discharge	Yes	66(13.9)
	No	410(86.1)
History of intravenous drug use	Yes	194(40.8)
	No	282(59.2)
Injectable medications	Yes	29(6.1)
	No	447(93.9)
Dental procedures	Yes	49(10.3)
	No	427(89.7)
Multiple sexual partners	Yes	46(9.7)
	No	430(90.3)
Unsafe sex	Yes	67(14.1)
	No	409(85.9)
Family history of hepatitis	Yes	25(5.3)
	No	451(94.7)
History of mortality due to hepatitis in family	Yes	25(5.3)
	No	451(94.7)
Stabbing, contact with possibly contaminated cutting objects	Yes	33(6.9)
	No	443(93.1)
Previous history of emigration	Yes	34(7.1)
	No	442(92.9)
History of sexually transmitted illness	Yes	84(17.6)
	No	392(82.4)
History of receiving traditional delivery care	Yes	105(22.1)
	No	371(77.9)
Presence of opportunistic infection	Yes	36(7.6)
	No	440(92.4)
Alcohol drinking	Yes	19(4)
	No	457(96)
Khat chewing	Yes	68(14.3)
	No	408(85.7)
Smoking Cigarettes	Yes	20(4.2)
	No	456(95.8)

Out of the 476 study participants, 252 (52.9%) had a history of ANC visits, and 340 (71.4%) had a history of previous deliveries, with 180 (52.9%) women having a history of institutional delivery. Additionally, 76 (16%) had a history of abortions, and 128 (26.9%) had female genital mutilation (FGM). Of the 340 women with a history of previous deliveries, 309 (64.9%) women had a history of two or more pregnancies (multigravida), and 238 (50%) women had never given birth (nulliparous). Furthermore, 51 (10.7%) of the women had a history of cesarean section (C/S). Two-thirds (316, 66.4%) of pregnant women were aware that HBV is transmitted sexually. Additionally, 345 (72.5%) pregnant women knew that HBV is transmitted through contact with blood and body fluids, and 364 (76.5%) pregnant women understood that HBV can be transmitted from mother to child (Table 3).

**Table 3:** Obstetric and clinical-related characteristics of the study participants.

		Total(n = 476)
Variables	Categories	n (%)
Have a previous history of ANC visits	Yes	252(52.9)
	No	224(47.1)
History previous delivery	Yes	340(71.4)
	No	136(28.6)
Place of the previous delivery(n = 340)	Home	160(41.1)
	Institution	180(52.9)
Previous history of abortion	Yes	76(16)
	No	400(84)
Female genital mutilation history	Yes	128(26.9)
	No	348(73.1)
Gravidity	Primigravida (1)	167(35.1)
	Multigravida (≥2)	309(64.9)
Parity	Nulliparous (0)	238(50)
	Primiparous (1)	114(23.9)
	Multiparous ((≥2)	124(26.1)
Previous caesarean section	Yes	51(10.7)
	No	425(89.3)
Is HBV transmitted sexually?	Yes(correct)	316(66.4)
	No	160(33.6)
Is HBV transmitted through contact with blood and body fluid?	Yes(correct)	345(72.5)
	No	131(27.5)
Is HBV transmitted from mother to child?	Yes(correct)	364(76.5)
	No	112(23.5)

Where: HBV is Hepatitis B virus, and ANC is antenatal care.

### 3.3. Seroprevalence of Hepatitis B virus infection

**Fig 2:**
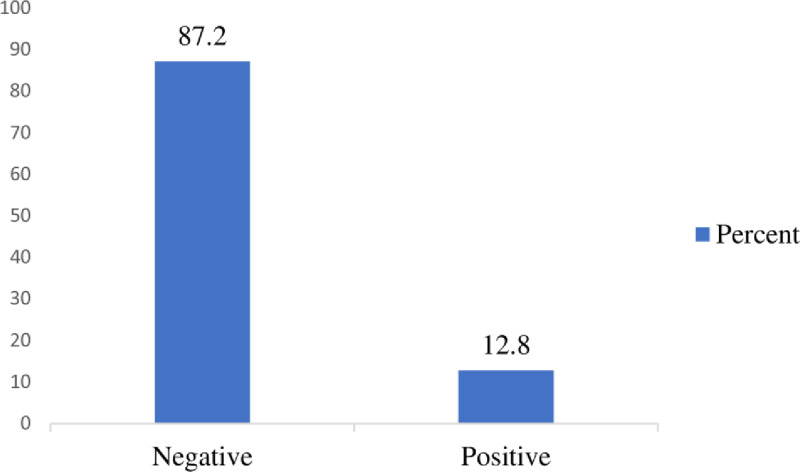
Seroprevalence of hepatitis B virus infection among pregnant women attending antenatal care clinics in public hospitals in Central Ethiopia region, 2023 (n = 476).

Overall, the seroprevalence of HBV infection among pregnant women was 12.8% (61/476) (95% CI, 10.1, 16.2) (Fig 2).

### 3.4. Factors associated with the prevalence of Hepatitis B virus infection

Our findings demonstrated that pregnant women with a diploma or higher educational attainment had 77% lower odds of being HBsAg-positive than those with primary and secondary education (AOR: 0.23; 95% CI: 0.09, 0.62). Similarly, those with no history of hospital admission also had a 77% lower odds of being HBsAg-positive than pregnant women with a previous history of hospital admission (AOR: 0.23; 95% CI: 0.10, 0.53), were associated with a reduced risk of HBV infection. Conversely, women unable to read and write had 2.67 times higher odds of being HBsAg-positive than those who had completed primary and secondary education (AOR: 2.67; 95% CI: 1.14, 6.26). Family size was also a significant factor: those from medium-sized families (4–6 members) had 2.34 times higher odds (AOR = 2.34; 95% CI: 1.15, 4.78), and those from large families (≥7 members) had 3.65 times higher odds (AOR = 3.65; 95% CI: 1.33, 10.04) of being HBsAg-positive compared to those from small families (1–3 members). In addition, a history of receiving traditional delivery care was associated with 2.46 times the higher odds of being HBsAg-positive than those with no history of receiving traditional delivery care (AOR: 2.46; 95% CI: 1.04, 5.84), and a history of abortion was associated with 2.90 times the higher odds of the HBsAg-positive than those no history of abortion (AOR: 2.90; 95% CI: 1.37, 6.11), were associated with an increased risk of HBV infection, with a p-value of less than 0.05 (Table 4).

**Table 4:** Factors associated with hepatitis B virus infection among pregnant women in the central Ethiopia region (n = 476).

Variable	HBsAg Status				
	Negative, n (%)	Positive, n (%)	COR (95%CI)	AOR (95%CI)	P-value
Educational level					
Unable to read and write	41(9.9)	21(34.4)	3.25(1.69,6.26) ^*^	2.67(1.14,6.26) ^**^	0.024
Primary and secondary education	184(44.3)	29(47.5)	1	1	
Certificate	79(19)	5(8.2)	0.40(0.15,1.08) ^*^	0.35(0.10,1.24)	0.103
Diploma and above	111(26.7)	6(9.8)	0.34(0.14,0.85) ^*^	0.23(0.09,0.62) ^**^	0.003
Family Size					
Small family (1–3)	232(55.9)	14(23)	1	1	
Medium family (4–6)	144(34.7)	32(52.5)	3.68(1.90,7.14) ^*^	2.34(1.15,4.78) ^**^	0.020
Large family (≥ 7)	39(9.4)	15(24.6)	6.37(2.85,14.23) ^*^	3.65(1.33,10.04) ^**^	0.012
History hospital admission					
Yes	235(56.6)	52(85.2)	1	1	
No	180(43.4)	9(14.8)	0.23(0.11,0.47) ^*^	0.23(0.10,0.53) ^**^	0.001
History of receiving traditional delivery care					
Yes	66(20.7)	19(31.1)	1.73(0.96,3.13) ^*^	2.46(1.04,5.84) ^**^	0.041
No	329(79.3)	42(68.9)	1		
Previous history of abortion					
Yes	57(13.7)	19(31.1)	2.84(1.54,5.23) ^*^	2.90(1.37,6.11) ^**^	0.005
No	358(86.3)	42(68.9)	1	1	

Where: COR = crude odds ratio, AOR = adjusted odds ratio, 1 = reference,

* P < 0.25 = variable candidate in bivariable analysis, and

** P < 0.05 = statistically significant in multivariable analysis.

## 4. Discussion

The findings indicate that the seroprevalence of HBV infection among pregnant women was 12.8% (95% CI, 10.1–16.2), which is classified as high according to the WHO criterion of HBsAg seroprevalence (≥8%) [37,45,51]. Significant factors associated with HBV infection included educational status (both diploma or higher, and unable to read and write), no history of hospital admission, medium-sized families (4–6 members) and large (≥ 7 members) family size, history of receiving traditional delivery care, and a history of abortion.

The seroprevalence of HBV infection observed in this study was higher than most previous findings reported across Ethiopia. In southern Ethiopia, studies have shown lower prevalence rates ranging from 3.3% to 9.2%, including areas such as the Silte Zone, Gurage Zone, Arba Minch, Borena, Hawassa, Wolaita, and Gedeo [9,46,52–57]. Similarly, studies from Babile, Hararghe, Jigjiga, and Dire Dawa in eastern Ethiopia reported lower rates, typically between 7.9% and 8.5% [41,44,58,59]. In central, western, and northern Ethiopia, even lower rates have been documented, such as in Addis Ababa (2.3–3%) [60,61], Jimma (3.1%) [62], Nekemte (5.8%) [63], Bahir Dar (4.5%) [64], and Amhara Region (4.6%) [65]. The studies from Dilla (11%) [66], Mogadishu, Somalia (11.2%) [67], Borumeda (11.3%) [25], Tigray region (11.6%) [37], and Debre Tabor (13%) [68] reported comparable rates. This variation in HBV prevalence in the Ethiopian region can be attributed to differences in cultural practice, access to health services, screening coverage, strategies for managing risk behaviors, and the study’s period.

Internationally, the prevalence reported in this study is also higher than those documented in countries like India (1.6%) [69], Angola (2.6%) [70], Tanzania (3.9%) [71], Nigeria (5.8-6.5%) [72,73], and Ghana (7.7%) [45]. These low rates are affected by strong national HBV screening programs, high vaccination coverage, and practical prevention work [55]. This variation may be attributed to differences in the health system’s performance in the design of HBV epidemiology in areas, sociodemographic context, and the role of public health interventions.

In the present study, pregnant women who were unable to read and write had higher odds of being HBsAg-positive compared to those who had completed primary and secondary education. Conversely, those with a diploma or higher educational attainment had lower odds of being HBsAg-positive than women with primary and secondary education. These findings suggest that higher educational attainment is associated with reduced odds of HBV infection. This is consistent with findings from studies conducted at Korle-Bu Teaching Hospital, Ghana [45], southern Ethiopia [56], Jimma in southwest Ethiopia [62], a systematic review and meta-analysis conducted in Nigeria [73], in India [69], and Mogadishu, Somalia [67]. Pregnant women with a higher level of education can raise awareness of health services, more significant health skills, and preventive measures such as antenatal screening [69]. Education can also serve as a proxy for the socio-economic situation, which can affect exposure to health-seeking behaviours and risk factors. These factors can help reduce the risk of HBV infection among more educated women.

Pregnant women from medium-sized families (4–6 members) and large families (≥ 7 members) had higher odds of being HBsAg-positive compared to those from small-sized families (1–3 members).These findings align with previous studies conducted in Gojjam zones in northwest Ethiopia [74], Debretabor Hospital in South Gondar-Northwest Ethiopia [75], and Addis Ababa, Ethiopia [76]. Other studies provide evidence that pregnant women with a large number of household members infected or exposed to HBV increase the risk of HBV transmission to family members [77]. The increased rate of HBV infection in larger families may be attributed to both horizontal and vertical transmission of the virus within the family unit. In larger households, there is a greater likelihood of close contact among family members, elevating the risk of HBV transmission. Additionally, larger families may experience financial constraints that hinder their ability to seek preventive care, such as vaccinations or timely medical attention for HBV-related conditions.

Pregnant women with no history of hospital admission had lower odds of being HBsAg-positive than women with a history of hospital admission. These findings are similar to previous studies conducted in various regions of Ethiopia, including the Eastern Zone of Tigray [37], Hawassa city public hospitals in Southern Ethiopia [46], Borena Zone in Southern Ethiopia [55], a previous systematic review and meta-analysis conducted in Ethiopia [78], Gedeo Zone in southern Ethiopia [57], the Eastern part of the Amhara region in Ethiopia [79], and West Hararghe in the Oromia region of Ethiopia [51]. However, this study’s findings contradict a study conducted at Agena Health Center in South Ethiopia [53]. This discrepancy may be attributed to insufficient infection prevention and control measures during hospital admissions. Hospitals, especially those with inadequate infection control practices, can be sources of nosocomial infections, including HBV [79]. Hospitalized women may be at an increased risk of acquiring the infection due to factors such as inadequate sterilization processes, cross-contamination, insufficient vaccination coverage for pregnant women against HBV, and a lack of proper bed hygiene between the discharge of HBsAg-positive patients and the admission of new patients. Furthermore, hospital admissions frequently involve invasive medical procedures, including injections, blood transfusions, surgeries, and the use of medical devices (e.g., catheters, IV lines).

Pregnant women with a history of receiving traditional delivery care had higher odds of being HBsAg-positive compared to those with no history of receiving traditional delivery care. These findings were similar to previous studies conducted in the eastern part of the Amhara region, Ethiopia [79], the Tigray Region, Northern Ethiopia [37], and Indonesia [80]. This could be due to several potential reasons related to traditional birth attendants (TBAs) lacking formal training and medical recognition; they hold a respected position in society and are often sought after as healthcare providers. TBAs are typically older women who have acquired their skills through apprenticeship or self-teaching [81]. This issue may be attributed to using non-sterilized instruments and materials, unsafe delivery techniques, and inadequate infection prevention measures, such as using gloves during traditional delivery care. Traditional delivery care providers often employ non-sterile instruments, increasing the risk of transmitting bloodborne infections, including Hepatitis B Virus (HBV). Reusing cutting tools, such as blades for umbilical cord cutting, without proper sterilization can exacerbate this problem. Furthermore, certain traditional practices may involve handling tissues in a manner that elevates the risk of HBV transmission.

Pregnant women with a history of abortion had higher odds of being HBsAg-positive compared to those with no history of abortion. These findings are consistent with a systematic review and meta-analysis conducted in various regions of Ethiopia [78], Gedeo Zone in southern Ethiopia [57], Nekemte town in western Ethiopia [63], West Hararghe in the Oromia region [51], Borumeda General Hospital in northeast Ethiopia [82], Jimma in southwest Ethiopia [62], and Arba Minch Hospital in south Ethiopia [54]. However, these findings contrast the study conducted at Adigrat General Hospital in northern Ethiopia [83] and Agena Health Center in south Ethiopia [53]. Since abortion in Ethiopia was liberalized in 2005, it allowed for a broader range of conditions under which it is legal. According to Ethiopian law, abortion is permitted in cases of rape, incest, or fetal impairment. It is also allowed when the pregnancy endangers the life of the woman or in cases where the woman is a minor and is physically or psychologically unprepared for childbirth [51,84,85]. This might be due to poor hygiene, contaminated instruments, and a lack of personal protective equipment, which can expose women to multiple infections during this procedure [45,48,59,63]. Also, abortion is frequently associated with unwanted and unsafe sexual intercourse, which in itself may increase the risk of HBV infection [51,54,57,62,78].

## 5. Strengths and limitation

The study’s strengths include using a standardized questionnaire, a large sample size, and blood screening for HBV (HBsAg) infections. However, several limitations exist. First, alternative methods such as molecular nucleic acid testing (NAT), hepatitis B surface antibody (anti-HBs), hepatitis B e antigen (HBeAg), and anti-hepatitis B core IgM (anti-HBc IgM) assays were not performed on HBsAg-positive samples due to limited resources. The study did not assess the chronicity of the virus or antibody levels in fully vaccinated adults, which would have confirmed immunity and excluded them from HBsAg testing. Furthermore, anti-HBc was not assessed to determine the actual viral burden and the incidence of occult infections. Analysis of the virus’s chronicity was not done.

Additionally, because the study was only done once, it cannot identify any causal relationships. Pregnant women who sought ANC services at private health facilities were not included in the study, which was conducted only in public health institutions. Moreover, social desirability bias may have occurred, leading participants to provide responses they believed to be more socially acceptable than accurate due to the potential stigma associated with being positive.

## 6. Conclusions and recommendations

When compared to other research, the seroprevalence of HBV infection among pregnant women in the central Ethiopia region was high, at 12.8%. Factors such as the maternal educational status (both diploma or higher, and unable to read and write), medium-sized families (4–6 members) and large (≥ 7 members) family size, no history of hospital admission, a history of receiving traditional delivery care, and a history of abortion were associated with HBV infection among pregnant women. It is recommended that all pregnant women with low educational levels use visual and oral methods to communicate information on HBV prevention, vaccination, and transmission.

In addition, the government should advocate for family-oriented health services that promote screening and vaccination for HBV among all household members, especially in more prominent families. Healthcare professionals should use programs to return home to identify pregnant women who have not accessed health facilities and ensure that they receive the necessary HBV testing, counselling, and education. Furthermore, it is recommended to develop training programs for traditional birth attendants, emphasizing HBV prevention strategies, sterilization techniques, and the importance of referring high-risk pregnancies to health facilities.

All women undergoing abortion should receive comprehensive post-abortion care, including HBV screening, counselling, and vaccination if they test negative. In addition, if a mother performs positive tests for HBV, it is necessary to ensure that the newborn receives the HBV vaccine within 24 hours of birth to prevent the transfer of the mother-child. Policy-makers have mandated the expansion of HBV screening beyond pregnant women to include their household members, particularly in high-prevalence areas. It is also necessary to strengthen the integration of HBV services into regular ANC and community Health platforms. Researchers should also study HBV vaccination and consciousness campaigns to interactively prevent and control HBV infection.

## Supporting information

S1 Text English version of the questionnaire. (DOCX)

S1 Data SPSS dataset. (SAV)

S1 Checklist STROBE Statement—Checklist of items. (DOCX)

S2 Checklist Inclusivity in global research. (DOCX)
