# Supplementary material for: Seroprevalence and associated factors of Hepatitis B virus infection among pregnant women attending Antenatal care clinic in public hospitals in the Central Ethiopia region: A cross-sectional study
Source: PLOS Glob Public Health. 2025 Jun 3;5(6):e0003921. doi: 10.1371/journal.pgph.0003921 (PMC12132948; doi:10.1371/journal.pgph.0003921)
Supplement: S1 Text — (DOCX) [file pgph.0003921.s001.docx]

**English Version Questioners**

**Consent Form and Information of the Study Participants**

**The project proposal title is** “Seroprevalence and Associated Factors of the hepatitis B virus infection among pregnant women receiving antenatal care services at public hospitals in the central Ethiopian region: A Cross-Sectional Study."

The Central University of Tamil Nadu, Department of Epidemiology and Public Health, Tamil Nadu, Thiruvarur, India.

Good morning/afternoon. Hello, how are you? My name is _______, and I am working as a data collector for Mr. Yilma Markos Larebo, a student of doctoral philosophy (Ph.D.) on an Indian Council for Cultural Relations (ICCR) scholarship at the Central University of Tamil Nadu (CUTN), department of epidemiology and public health (EPH), under the supervisor Natarajan Gopalan (Ph.D., Professor) in Tamil Nadu, Thiruvarur, India. This study aims to assess Seroprevalence and Associated Factors of the hepatitis B virus infection among pregnant women receiving antenatal care services at public hospitals in the central Ethiopian region. The blood samples will be taken from pregnant mothers and cord of the new-born baby in the hospital laboratory. The study participants' privacy confidentiality, and none of the participants paid for the test will be considered. All women who test positive for HBsAg will be counselled on their status, disease transmission routes, the need for neonatal immunization, and close-contact hepatitis screening. They will then be referred to internal medicine linkage for prevention, treatment, and care services.

The study findings will also assist programme managers and advocates in developing and implementing evidence-based interventions and establish community-based screening programs. It could serve as the foundation for future research into health education, promotion, and implementation. The study's findings will fill research gaps by assisting the community in raising awareness about viral hepatitis surveillance, prevention, control, risk factors, and affected bodies and taking appropriate actions. Furthermore, the findings will be used as a reference for those researching the same topic.

I will ask you some questions concerning the risk of HBV infection on seroprevalence, mother-to-child transmission and associated factors of the hepatitis B virus among pregnant Mothers at delivery point Central Ethiopia Region. It will take about 15 to 45 minutes. The information you provide us is confidential and will not be shared with anyone else. Your name or any identifying information will not be registered.

You may refuse to answer any question and choose to stop the interview at any time. The information you provide us is essential and valuable, as it will help the government and the health facilities involved in the treatment of HBV and give awareness to the community to improve service delivery.

Name of Investigator: Mr. Yilma Markos Larebo; Cell Phone: +251-917-189-182; Email: yilmamark@gmail.com.

Do I have your permission to continue? 1: Yes; 2: No;

If the answer is yes, thank you and conduct the interview.

If the answer is no, give thanks and proceed to the next respondent's mother.

Zone/Special woreda Name: ___________________: Name of the hospital: _______________

Medical Record Number (MRN): _______________: Date of interview: _________________ Time started: ___________________: Interviewer Signature: __________________________

Respondent Signature: _____________Supervisor's name: __________________________ Supervisor’s Signature: ___________________Time ended: __________________________

**English Version Questionnaires**

Table 1: Socio-demographic related characteristics of study participants on seroprevalence and Associated Factors of the hepatitis B virus infection among pregnant women receiving antenatal care services at public hospitals in the central Ethiopian region: A Cross-Sectional Study, 2023.

| **S. No** | **Questionnaire and filters** | **Categories** | **Code** |
| --- | --- | --- | --- |
| Q1 | Age in completed years | ______________ | 1 |
| Q2 | Residence | Urban  Rural | 1  2 |
| Q3 | What is your marital status now? | Single  Married  Divorced  Widowed  Separated | 1  2  3  4  5 |
| Q4 | What is your religion? | Orthodox  Muslim  Protestant  Catholic  I have no religion  Others­­­­_________ | 1  2  3  4  5  6 |
| Q5 | What is your educational status now? | Unable to read and write  Primary and secondary education  Certificate  Diploma  Degree and above | 1  2  3  4  5 |
| Q6 | What is your occupation status now? | Civil servant  Self-employers  Merchant  Farmer  Housewife  Sex worker  Student  Military  Other___________________ | 1  2  3  4  5  6  7  8  9 |
| Q7 | Family size | ___________ | 1 |
| Q8 | What is your ethnicity? | Hadiya  Kembata  Gurage  Silte  Halaba  Yem  Amhara  Wolaita  Oromo  Tigre  Others specify___________ | 1  2  3  4  5  6  7  8  9  10  11 |
| Q9 | Do you have information about HBV infection? | Yes  No | 1  2 |
| Q10 | If yes, in Q9, what are the sources of information about HBV infection? | Television/ Radio  Newspapers/magazines  Health workers/ Health care providers  Social media  Their friends/ Family /friends/neighbour  Religious leaders/teachers  HBV info. leaflets/ brochures/ posters  Other sources specify _________ | 1  2  3  4  5  6  7  8 |
| Q11 | Average monthly income______ | ___________ETB | 1 |
| Q12 | HbsAg (**Hepatitis B surface antigen**) | Positive  Negative | 1  0 |

Where HBV: Hepatitis B Virus; ANC: Antenatal care; ETB: Ethiopian Total Birr

Table 2: Past medical history and behavioral risk-related characteristics of study participants on seroprevalence and Associated Factors of the hepatitis B virus infection among pregnant women receiving antenatal care services at public hospitals in the central Ethiopian region, 2023. Please give an appropriate answer.

| **S. No** | **Questionnaire and filters** | **Categories** | **Code** |
| --- | --- | --- | --- |
| Q13 | History hospital admission | Yes  No | 1  2 |
| Q14 | History of day-care /hospital stay | Yes  No | 1  2 |
| Q15 | History of surgery | Yes  No | 1  2 |
| Q16 | Tonsillectomy | Yes  No | 1  2 |
| Q17 | Phlebotomy | Yes  No | 1  2 |
| Q18 | History of blood donation | Yes  No | 1  2 |
| Q19 | If yes in question number 18, the donation type | Voluntary  Replacement | 1  2 |
| Q20 | Frequency of donation | First time  Repeated | 1  2 |
| Q21 | Tattooing | Yes  No | 1  2 |
| Q22 | Body piercing | Yes  No | 1  2 |
| Q23 | Genital discharge | Yes  No | 1  2 |
| Q24 | History of intravenous drug use or injectable medications | Yes  No | 1  2 |
| Q25 | Non-IV drug abuser | Yes  No | 1  2 |
| Q26 | Tooth extraction/dental procedures | Yes  No | 1  2 |
| Q27 | Multiple sexual partners/ extramarital intercourse | Yes  No | 1  2 |
| Q28 | Unsafe Sex (without condom) | Yes  No | 1  2 |
| Q29 | Family history of liver disease(hepatitis) | Yes  No | 1  2 |
| Q30 | Previous history of contact with hepatitis patients | Yes  No | 1  2 |
| Q31 | History of hepatitis family (maternal and paternal). | Yes  No | 1  2 |
| Q32 | History of mortality due to hepatitis in family | Yes  No | 1  2 |
| Q33 | Others (stabbing, contact with possibly contaminated cutting objects) | Yes  No | 1  2 |
| Q34 | Previous history of emigration | Yes  No | 1  2 |
| Q35 | History of sexually transmitted illness (STI) | Yes  No | 1  2 |
| Q36 | Provision of traditional delivery care | Yes  No | 1  2 |
| Q37 | Presence of opportunistic infection | Yes  No | 1  2 |
| Q38 | HIV serostatus | Positive  Negative | 1  2 |
| Q39 | Alcohol consumption/drinking | Yes  No | 1  2 |
| Q40 | Chat chewing | Yes  No | 1  2 |
| Q41 | Smoking Cigarettes | Yes  No | 1  2 |

Where: HIV: Human Immune Virus, STI: Sexually Transmitted Illness.

Table 3: Obstetric and clinical-related characteristics of the study participants on seroprevalence and Associated Factors of the hepatitis B virus infection among pregnant women receiving antenatal care services at public hospitals in the central Ethiopian region, 2023. Please give an appropriate answer.

| **S. No** | **Questionnaire and filters** | **Categories** | **Code** |
| --- | --- | --- | --- |
| Q42 | Have pervious history of ANC visits | Yes  No | 1  2 |
| Q43 | History of pervious delivery | Yes  No | 1  2 |
| Q44 | Place of the previous delivery (from total previous delivered pregnant women in question 44) | Home  Health institution | 1  2 |
| Q45 | Previous history of abortion | Yes  No | 1  2 |
| Q46 | Female genital mutilation (FGM) history | Yes  No | 1  2 |
| Q47 | Gestational age | _______________ | 1 |
| Q48 | Gravidity | Primigravida (1)  Multigravida (≥2) | 1  2 |
| Q49 | Parity | Nulliparous (0)  Primiparous (1)  Multiparous ((≥2) | 1  2  3 |
| Q50 | Is HBV transmitted sexually? | Yes(correct)  No | 1  2 |
| Q51 | Is HBV transmitted through contact with blood and body fluid? | Yes(correct)  No | 1  2 |
| Q52 | Is HBV transmitted from mother to child? | Yes(correct)  No | 1  2 |
| Q53 | Other routes of transmission mentioned | ___________________ | 1 |

Where: HBV is Hepatitis B virus and ANC is antenatal care.
